# Supplementary material for: Exploring the cecal microbial community associated with fat deposition in sheep and its possible pathways of action
Source: Microbiol Spectr. 2025 May 22;13(7):e01488-24. doi: 10.1128/spectrum.01488-24 (PMC12210990; doi:10.1128/spectrum.01488-24)
Supplement: Supplemental material — Fig. S1 to S4; Tables S1 to S6. [file spectrum.01488-24-s0001.docx]

**Supplementary material**

Supplementary Figure S1. (a) Species accumulation curve. (b) Dilution curve based on Sobs index.


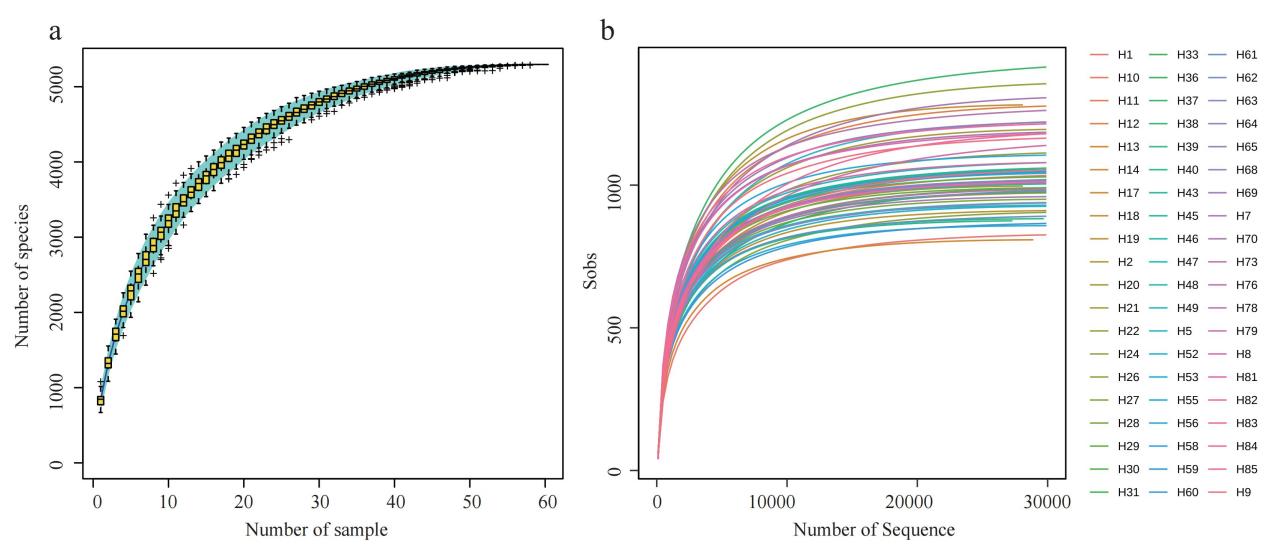


Supplementary Figure S2. Permutation testing of the random forest model.


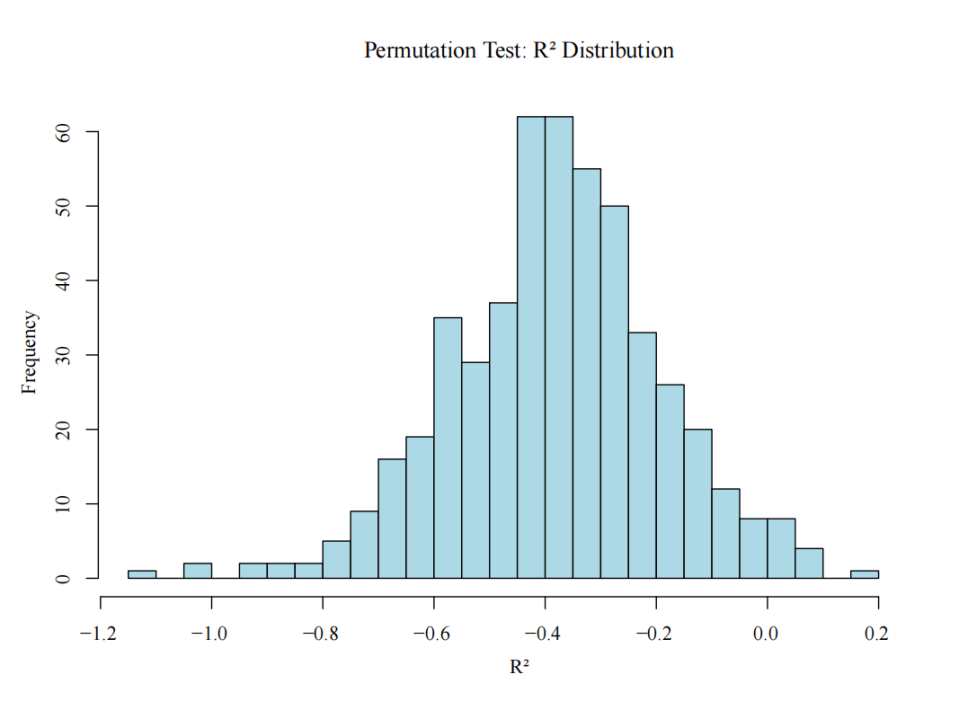


Supplementary Figure S3. SIMPER analysis between groups. (a) LB-HB. (b) LB-MB. (C) MB-HB.


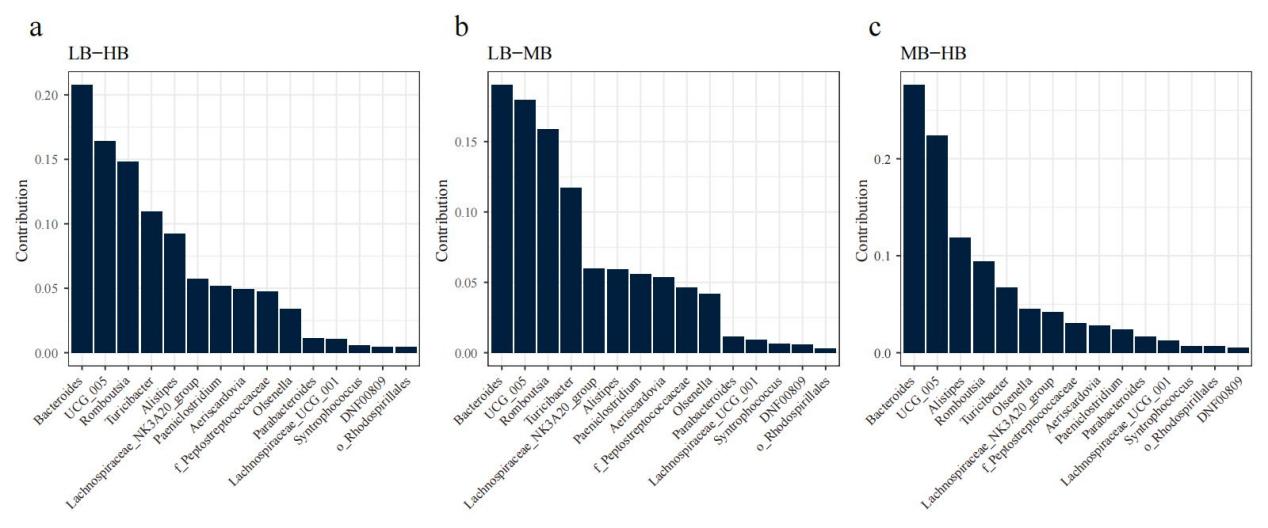


Supplementary Figure S4. Pearson correlation heatmap of biomarkers and fat deposition traits.


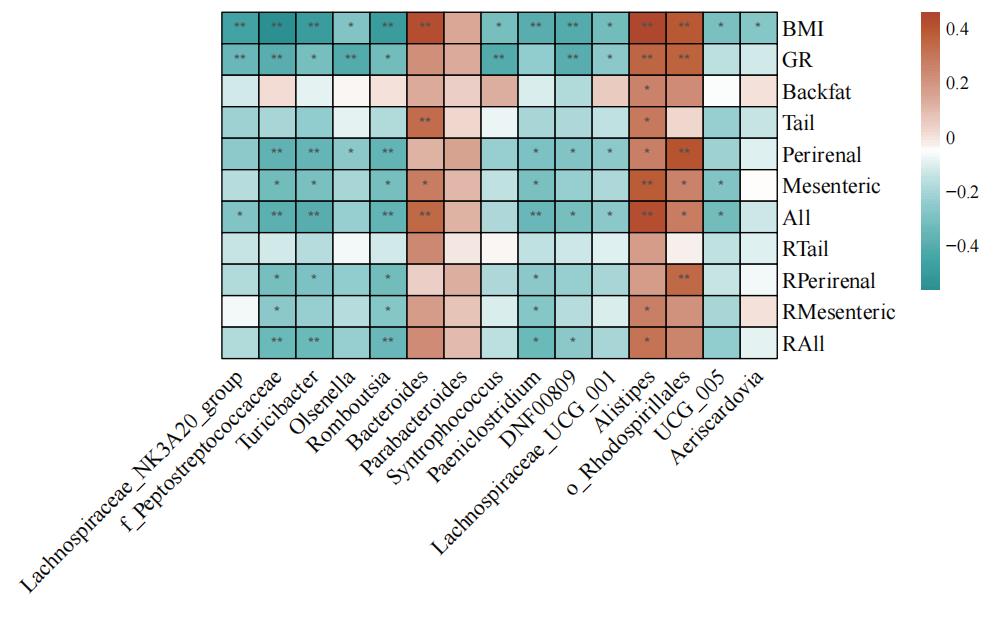


Note: *P* < 0.05 indicates statistical significance. * *P* < 0.05, ** *P* < 0.01.

Supplementary Table S1. Complete price pellet feed formula.

| project | item | Value |
| --- | --- | --- |
| Ingredient Composition | Corn [%] | 32.5 |
|  | Corn germ meal [%] | 18 |
|  | Corn stalks [%] | 12 |
|  | Corn hulls [%] | 11.2 |
|  | Corn cob [%] | 8 |
|  | Soybean meal [%] | 5 |
|  | Cotton meal [%] | 5 |
|  | Molasses [%] | 3.3 |
|  | Bentonite [%] | 1.5 |
|  | Baking soda [%] | 1 |
|  | Stone powder [%] | 0.8 |
|  | Expanded Urea [%] | 0.5 |
|  | Premix [%] | 0.5 |
| Chemical Composition | Dry matter (DM) [%] | 88.78 |
|  | Crude protein (CP) [%] | 13.09 |
|  | Digestible energy [MJ/kg] | 11.11 |
|  | Neutral detergent fiber (NDF) [%] | 27.08 |
|  | Acid detergent fiber (ADF) [%] | 13.99 |
|  | Crude fiber (CF) [%] | 9.78 |

Supplementary Table S2. Descriptive statistics of the experimental population.

| Characteristics | All (n = 303) | LB (n = 20) | MB (n = 20) | HB (n = 20) |
| --- | --- | --- | --- | --- |
| BMI, kg/m2 | 85.28±7.57 | 73.52±3.26 | 84.47±0.38 | 99.92±3.58 |
| GR, cm | 1.45±0.4 | 1.17±0.38 | 1.32±0.4 | 1.82±0.43 |
| Backfat, cm | 0.55±0.21 | 0.54±0.2 | 0.52±0.23 | 0.7±0.29 |
| Tail, kg | 1.1±0.38 | 0.92±0.29 | 1.1±0.28 | 1.54±0.57 |
| Perirenal, kg | 0.65±0.33 | 0.54±0.3 | 0.6±0.23 | 0.9±0.4 |
| Mesenteric, kg | 0.99±0.38 | 0.77±0.31 | 0.97±0.28 | 1.29±0.46 |
| All, kg | 2.74±0.83 | 2.24±0.7 | 2.67±0.58 | 3.72±0.79 |
| RTail | 0.02±0.01 | 0.02±0.01 | 0.02±0.01 | 0.03±0.01 |
| RPerirenal | 0.01±0.01 | 0.01±0.01 | 0.01±0 | 0.02±0.01 |
| RMesenteric | 0.02±0.01 | 0.02±0.01 | 0.02±0.01 | 0.02±0.01 |
| RAll | 0.06±0.01 | 0.05±0.01 | 0.06±0.01 | 0.07±0.01 |

Supplementary Table S3. Overview of 16S rDNA data from sheep cecum samples.

| Sample Name | Raw Tags | Effective Tags | AvgLen(nt) | Q20 | Q30 | GC% |
| --- | --- | --- | --- | --- | --- | --- |
| Hu1 | 70,447 | 55,563 | 413 | 97.74 | 93.11 | 53.40 |
| Hu2 | 69,467 | 55,697 | 414 | 97.81 | 93.25 | 53.20 |
| Hu3 | 75,568 | 60,155 | 413 | 97.79 | 93.18 | 52.81 |
| Hu4 | 74,962 | 58,123 | 413 | 97.77 | 93.11 | 52.76 |
| Hu5 | 72,953 | 55,368 | 412 | 97.73 | 93.14 | 53.19 |
| Hu6 | 67,919 | 54,344 | 409 | 97.76 | 93.20 | 53.35 |
| Hu7 | 74,876 | 57,693 | 408 | 97.83 | 93.33 | 53.61 |
| Hu8 | 62,787 | 49,163 | 410 | 97.68 | 92.93 | 53.51 |
| Hu9 | 68,962 | 49,387 | 411 | 97.64 | 92.92 | 53.60 |
| Hu10 | 74,127 | 53,875 | 412 | 97.63 | 92.87 | 53.07 |
| Hu11 | 72,484 | 58,133 | 412 | 97.59 | 92.83 | 53.25 |
| Hu12 | 75,694 | 54,816 | 413 | 97.65 | 92.71 | 53.28 |
| Hu13 | 67,284 | 53,471 | 409 | 97.72 | 93.09 | 53.28 |
| Hu14 | 69,737 | 50,038 | 411 | 97.68 | 92.96 | 53.11 |
| Hu15 | 64,072 | 50,226 | 410 | 97.48 | 92.35 | 53.56 |
| Hu16 | 83,612 | 60,462 | 408 | 97.60 | 92.82 | 53.64 |
| Hu17 | 71,158 | 51,957 | 410 | 97.72 | 93.05 | 53.19 |
| Hu18 | 78,451 | 58,075 | 410 | 97.68 | 92.91 | 53.10 |
| Hu19 | 59,597 | 48,123 | 413 | 97.69 | 92.93 | 52.94 |
| Hu20 | 69,721 | 50,601 | 411 | 97.63 | 92.86 | 53.08 |
| Hu21 | 71,362 | 52,385 | 410 | 97.76 | 93.12 | 53.16 |
| Hu22 | 73,306 | 55,917 | 412 | 97.65 | 92.84 | 53.67 |
| Hu23 | 69,685 | 52,534 | 413 | 97.85 | 93.24 | 53.43 |
| Hu24 | 68,348 | 52,243 | 410 | 97.58 | 92.64 | 53.00 |
| Hu25 | 79,515 | 57,918 | 411 | 97.72 | 93.02 | 53.14 |
| Hu26 | 69,723 | 49,076 | 408 | 97.61 | 92.85 | 53.25 |
| Hu27 | 64,537 | 49,837 | 410 | 97.78 | 93.16 | 53.57 |
| Hu28 | 72,690 | 54,685 | 411 | 97.73 | 93.07 | 53.17 |
| Hu29 | 62,671 | 51,389 | 413 | 97.77 | 93.11 | 52.73 |
| Hu30 | 76,800 | 56,885 | 410 | 97.70 | 93.04 | 52.89 |
| Hu31 | 83,427 | 62,365 | 411 | 97.67 | 92.95 | 52.78 |
| Hu32 | 65,648 | 52,038 | 409 | 97.79 | 93.22 | 53.63 |
| Hu33 | 63,977 | 47,722 | 412 | 97.55 | 92.62 | 52.73 |
| Hu34 | 74,014 | 60,548 | 409 | 97.62 | 92.81 | 53.09 |
| Hu35 | 72,600 | 51,096 | 413 | 97.50 | 92.56 | 52.81 |
| Hu36 | 71,544 | 51,603 | 413 | 97.59 | 92.72 | 52.58 |
| Hu37 | 63,287 | 48,684 | 414 | 97.48 | 92.41 | 53.53 |
| Hu38 | 68,881 | 54,310 | 412 | 97.63 | 92.78 | 53.17 |
| Hu39 | 73,076 | 57,254 | 411 | 97.68 | 92.91 | 52.59 |
| Hu40 | 73,623 | 53,932 | 412 | 97.75 | 93.08 | 52.94 |
| Hu41 | 65,885 | 50,992 | 414 | 97.78 | 93.08 | 52.56 |
| Hu42 | 65,524 | 53,879 | 414 | 97.55 | 92.66 | 52.62 |
| Hu43 | 72,624 | 55,283 | 412 | 97.69 | 92.90 | 52.95 |
| Hu44 | 62,820 | 48,145 | 412 | 97.68 | 92.92 | 52.47 |
| Hu45 | 69,452 | 54,396 | 411 | 97.63 | 92.89 | 52.48 |
| Hu46 | 70,177 | 55,428 | 412 | 97.64 | 92.88 | 52.55 |
| Hu47 | 80,244 | 59,566 | 408 | 97.78 | 93.28 | 53.40 |
| Hu48 | 91,716 | 66,675 | 411 | 97.76 | 93.20 | 53.59 |
| Hu49 | 80,138 | 57,938 | 412 | 97.73 | 93.03 | 52.78 |
| Hu50 | 68,926 | 55,615 | 414 | 97.60 | 92.76 | 52.60 |
| Hu51 | 69,958 | 55,995 | 412 | 97.77 | 93.18 | 52.67 |
| Hu52 | 80,508 | 63,659 | 410 | 97.76 | 93.20 | 53.14 |
| Hu53 | 81,520 | 57,947 | 412 | 97.61 | 92.89 | 52.89 |
| Hu54 | 75,788 | 57,019 | 410 | 97.73 | 93.07 | 52.72 |
| Hu55 | 83,355 | 64,342 | 412 | 97.71 | 93.06 | 52.67 |
| Hu56 | 79,047 | 58,906 | 411 | 97.70 | 93.02 | 52.73 |
| Hu57 | 74,985 | 57,919 | 412 | 97.67 | 92.94 | 52.42 |
| Hu58 | 74,203 | 58,583 | 414 | 97.74 | 93.11 | 52.75 |
| Hu59 | 77,399 | 57,977 | 413 | 97.55 | 92.70 | 53.12 |
| Hu60 | 65,311 | 50,749 | 411 | 98.28 | 94.30 | 53.46 |

Supplementary Table S4. Analysis of differences in phylum and family among different groups.

| Phylum | LB | MB | HB |
| --- | --- | --- | --- |
| Firmicutes | 0.6746±0.0719^a^ | 0.6416±0.0675^ab^ | 0.6127±0.0802^b^ |
| Bacteroidota | 0.2003±0.0482^b^ | 0.2361±0.048^ab^ | 0.2746±0.0701^a^ |
| Spirochaetota | 0.0172±0.0143 | 0.0321±0.0276 | 0.0306±0.0289 |
| Verrucomicrobiota | 0.0285±0.0375 | 0.0199±0.016 | 0.0217±0.0229 |
| Desulfobacterota | 0.0215±0.0141 | 0.0197±0.0103 | 0.0211±0.015 |
| Firmicutes/Bacteroidota | 3.6187±1.21^a^ | 2.8989±0.9961^ab^ | 2.4641±1.1107^b^ |
| Family | LB | MB | HB |
| Lachnospiraceae | 0.1601±0.0423 | 0.1507±0.0295 | 0.1362±0.028 |
| Oscillospiraceae | 0.1302±0.0188 | 0.1216±0.0201 | 0.1187±0.0233 |
| Eubacterium | 0.0667±0.0213 | 0.0823±0.037 | 0.076±0.0263 |
| Rikenellaceae | 0.0554±0.0226^b^ | 0.0789±0.0303^a^ | 0.0858±0.0358^a^ |
| Prevotellaceae | 0.0624±0.0447 | 0.0663±0.0419 | 0.0739±0.0559 |
| Bacteroidaceae | 0.0379±0.0126^b^ | 0.05±0.0229^ab^ | 0.0593±0.0197^a^ |
| UCG-010 | 0.0389±0.0094 | 0.0459±0.0101 | 0.0455±0.0141 |
| Ruminococcaceae | 0.044±0.0092 | 0.042±0.0067 | 0.0441±0.0123 |
| Christensenellaceae | 0.0467±0.0122^a^ | 0.0429±0.0133^ab^ | 0.0369±0.0094^b^ |
| Monoglobaceae | 0.0405±0.0103 | 0.0434±0.0104 | 0.0421±0.0157 |

Note: Statistical data are expressed as mean ± standard deviation. *P* < 0.05 indicates statistical significance. Different lowercase letters in the same row indicate significant differences between different group (*P* < 0.05).

Supplementary Table S5. Analysis of differences in VFAs and blood biochemical indicators among different groups.

| Item | LB | MB | HB |
| --- | --- | --- | --- |
| Acetic acid% | 0.57±0.07 | 0.56±0.05 | 0.54±0.04 |
| Propionic acid% | 0.23±0.03 | 0.24±0.03 | 0.23±0.03 |
| Isobutyric acid% | 0.03±0.01^a^ | 0.02±0.01^b^ | 0.02±0.01^b^ |
| Butyrate% | 0.1±0.03 | 0.1±0.03 | 0.12±0.02 |
| Isovaleric acid% | 0.02±0.01 | 0.02±0.01 | 0.02±0.01 |
| Valeric acid% | 0.01±0.01 | 0.01±0 | 0.01±0.01 |
| ALB | 22.03±3.48 | 21.01±4.2 | 21.27±5.91 |
| ALP | 234.91±66.32 | 223.65±71.83 | 186.85±56.29 |
| ALT | 7.34±2.61 | 6.28±4.36 | 7.02±4.79 |
| AST | 80.85±12.56 | 76.39±21.58 | 80.09±28.08 |
| CK | 166.7±50.72 | 181.12±81.66 | 212.96±123.37 |
| CR | 32.04±5.03 | 31.57±7.74 | 31.25±7.44 |
| DBIL | 0.82±0.29 | 0.83±0.25 | 0.97±0.4 |
| GLU | 8.62±2.07 | 9.07±1.15 | 8.83±1.21 |
| LDH | 409.98±90.8 | 385.38±106.86 | 436.2±150.64 |
| TBIL | 0.51±0.26 | 0.49±0.21 | 0.44±0.3 |
| TG | 0.12±0.04^b^ | 0.13±0.03^ab^ | 0.15±0.03^a^ |
| TP | 58.95±7.87 | 53.5±11.69 | 54.17±16.2 |

Note: Statistical data are expressed as mean ± standard deviation. *P* < 0.05 indicates statistical significance. Different lowercase letters in the same row indicate significant differences between different group (*P* < 0.05).

Supplementary Table S6. Analysis of the mediating effect of *Lachnospiraceae_NK3A20_group* and BMI.

| Model effect | Effect | Boot SE | BootLLCI | BootULCI | Effect ratio |
| --- | --- | --- | --- | --- | --- |
| Total effect | -799.35 | 202.42 | -1204.55 | -394.16 | 100% |
| Direct effect | -684.73 | 198.22 | -1081.67 | -287.80 | 85.66% |
| Indirect effect | -114.62 | 104.50 | -393.80 | -0.97 | 14.34% |

Note: The indirect effect is *Lachnospiraceae_NK3A20_group* - TG -BMI.
